# Machine learning identifies ferroptosis-related gene ANXA2 as potential diagnostic biomarkers for NAFLD

**DOI:** 10.3389/fendo.2023.1303426

**Published:** 2023-12-19

**Authors:** Jingtong Qin, Peng Cao, Xuexuan Ding, Zeyao Zeng, Liyan Deng, Lianxiang Luo

**Affiliations:** ^1^ The First Clinical College, Guangdong Medical University, Zhanjiang, China; ^2^ Department of Pharmacy, Union Hospital, Tongji Medical College, Huazhong University of Science and Technology, Wuhan, China; ^3^ The Marine Biomedical Research Institute, Guangdong Medical University, Zhanjiang, China; ^4^ The Marine Biomedical Research Institute of Guangdong Zhanjiang, Zhanjiang, China

**Keywords:** NAFLD, ferroptosis, ANXA2, machine learning, time series analysis, typing

## Abstract

**Introduction:**

Non-alcoholic fatty liver disease (NAFLD), a major cause of chronic liver disease, still lacks effective therapeutic targets today. Ferroptosis, a type of cell death characterized by lipid peroxidation, has been linked to NAFLD in certain preclinical trials, yet the exact molecular mechanism remains unclear. Thus, we analyzed the relationship between ferroptosis genes and NAFLD using high-throughput data.

**Method:**

We utilized a total of 282 samples from five datasets, including two mouse ones, one human one, one single nucleus dataset and one single cell dataset from Gene Expression Omnibus (GEO), as the data basis of our study. To filter robust treatment targets, we employed four machine learning methods (LASSO, SVM, RF and Boruta). In addition, we used an unsupervised consensus clustering algorithm to establish a typing scheme for NAFLD based on the expression of ferroptosis related genes (FRGs). Our study is also the first to investigate the dynamics of FRGs throughout the disease process by time series analysis. Finally, we validated the relationship between core gene and ferroptosis by *in vitro* experiments on HepG2 cells.

**Results:**

We discovered ANXA2 as a central focus in NAFLD and indicated its potential to boost ferroptosis in HepG2 cells. Additionally, based on the results obtained from time series analysis, ANXA2 was observed to significantly define the disease course of NAFLD. Our results demonstrate that implementing a ferroptosis-based staging method may hold promise for the diagnosis and treatment of NAFLD.

**Conclusion:**

Our findings suggest that ANXA2 may be a useful biomarker for the diagnosis and characterization of NAFLD.

## 1 Introduction

Non-alcoholic fatty liver disease (NAFLD), a liver condition, results from the buildup of fat in the liver, unrelated to alcohol consumption. Depending on its severity, it can be classified as hepatic steatosis, nonalcoholic steatohepatitis (NASH), or cirrhosis (a stage that is difficult to reverse) (1). According to statistics, about 30-40% of patients eventually progress to cirrhosis, even up to 55% in some reports (2, 3). Not only that, NAFLD is currently one of the major causes of chronic liver disease worldwide, accounting for between 73% to 95% of all cases. A recent research in China suggests it may be surpassing hepatitis B as the primary cause of chronic liver disease (4). Without exaggeration, treating NAFLD will be a large and daunting proposition.

However, the threat of NAFLD lies not only in its high prevalence, but also in its complex and ill-defined pathogenesis. To date, there are no specific drugs approved to treat NAFLD and its advanced forms, although several potential drugs have been extensively studied in the last decades (5–7). This means that we still urgently need to identify more specific therapeutic targets. Moreover, the staging methods for NAFLD are not well established. Despite significant differences between the different staging, reliable *in vitro* diagnostic tools to distinguish fatty liver from NASH are still lacking, and invasive liver biopsy remains the main diagnostic method (8). In the search for solutions to these problems, several studies have focused on the large accumulation of lipid peroxides in the pathogenesis of NAFLD (9). Combining this feature with the fact that iron overload is prevalent in patients, people link the disease to the concept of ferroptosis. Some evidence that has emerged gives confidence in targeting ferroptosis to treat NAFLD.

Ferroptosis, a newly discovered form of cell death, distinguished by massive iron accumulation and lipid peroxidation (10). A growing number of studies suggest that it is closely associated with NAFLD. We cite several studies that provide evidence supporting this association, including 1) Hernández-Aguilera et al. showed that increased iron content in hepatocytes leads to fat accumulation, causing cellular damage and exacerbating NAFLD (11); 2) a human liver specimen study reporting a positive correlation between ferroptosis in hepatocytes of NAFLD patients and the degree of hepatocellular pathological damage (12); 3) using iron chelators attenuated hepatitis in a mouse model of NAFLD and fibrosis (13); 4) ferroptosis and mitochondrial damage are major factors in the development of NAFLD, and by inhibiting ferroptosis, mitochondrial damage and inflammatory responses can be reduced, thus improving hepatocyte function and injury (14). Based on the above, we can say that ferroptosis has great potential in the study of NAFLD. However, studies based on high-throughput data analyzing the impact of ferroptosis molecules on NAFLD are not available in today’s scientific field, suggesting that the role and mechanisms of the sizeable set of ferroptosis genes in the pathogenesis of NAFLD are still unknown. Therefore, we hope to find reliable ferroptosis targets and provide important clues for more studies at the molecular level.

In this study, we combined mouse, human and single cell sequencing datasets to investigate the role of ferroptosis molecules in NAFLD, a total of 282 samples were included. Additionally, we used four machine learning methods (LASSO, SVM, RF and Boruta) to find the most robust target, resulting in ANXA2, a ferroptosis target that has never been mentioned before. As mentioned earlier, NAFLD is a multi-stage disease. We used time series to analyze the dynamics of ferroptosis molecules in it, which is rarely seen and necessary. Finally, we performed cellular experiments to verify the relationship between core genes and ferroptosis by assaying indicators including ferrous ions, mitochondria and reactive oxygen species and oxidative metabolites.

## 2 Materials and methods

### 2.1 Collection and pre-processing of disease and health samples

We retrieved the data for the study from the Gene Expression Omnibus database (GEO) (15), applying the following criteria: 1) search results using the keywords “NAFLD and NASH”; 2) sample size greater than 30; 3) sampling sites limited to liver tissues; 4) mouse samples having time sequences and human samples having patients with different fibrosis stages. Three RNA sequencing data sets were selected, namely GSE40481 and GSE109345, which are mouse-derived, and GSE162694, which is human-derived (**Supplementary Table S1**). Out of these, GSE40481 served as the training set, comprising of 27 control samples and 24 high-fat diet-induced NASH samples. GSE109345 was designated as the validation set, which had 30 control samples and 48 samples with NASH. Last but not least, GSE162694 was assigned as the human validation set, with 66 NAFLD samples having no liver fibrosis (normal/fibrosis stage = 0) and 77 NAFLD samples with varying degrees of liver fibrosis (fibrosis stage = 1/2/3/4). In addition, GSE158241 is a single-cell sequencing data which incorporates liver biopsy samples from four healthy mice and two NASH mice. GSE225381, a single-nucleus sequencing dataset, which included two control samples and two NAFLD samples. (**Supplementary Table S1**). To ensure uniformity across the gene sets, we used the R package “homologene” to transform the mouse gene set into the human homologene set. We also removed batch effects between the data sets to increase the effectiveness of our findings, using the sva package’s combat function. Principal component analysis (PCA) method was used to evaluated the performance of the combat function (**Supplementary Figure S1**).

### 2.2 Acquisition of ferroptosis gene sets

We obtained sets of genes associated with ferroptosis, including drivers, suppressors, and markers, from FerrDb V1 (http://www.zhounan.org/ferrdb/), and intersected them with search results in Genecard (https://www.genecards.org/) using “ferroptosis” as a keyword. All non-protein coding genes were excluded. Finally, we identified 804 protein-coding genes related to ferroptosis, which we named “FRGs”.

### 2.3 Screening module genes by weighted gene co-expression network analysis

The “WGCNA” in R was used to construct an unsupervised co-expression network and identify the gene modules associated with NAFLD. Ferroptosis gene expression was normalized and hybrid robust-Pearson correlation coefficient formula was utilized to determine the relationship between gene expression and NAFLD. A weighted adjacency matrix and transformed topological overlap matrix (TOM) were constructed, followed by filtering cells containing more than 50 genes by a stratified clustering tree approach. Distinct branches of the clustering indicate different gene modules, with a high degrees of gene co-expression present within modules. The highly correlated modules comprise genes that strongly relate to the targeted disease (16).

### 2.4 Differential expression analysis of genes

The limma package was used for differential analysis of control and NASH samples in GSE40481, with criteria of |Log2fold change|>1 and P value<0.05, identifying differentially expressed genes (DEGs). Subsequently, volcano maps of ANXA2 and other DEGs were plotted by the R package “ggVolcano”.

### 2.5 Identification of disease signature genes

To perform feature selection of disease diagnostic factors, we incorporated a combination of powerful machine learning algorithms, namely LASSO, SVM, Boruta, and RF, to identify NAFLD signature genes. The Boruta algorithm, a supervised classification feature selection method, was utilized to accurately pinpoint all relevant features. For categorical variables, the least absolute shrinkage and selection operator (LASSO) was used, as it has been proven to effectively improve statistical model predictability and interpretability. Support Vector Machine (SVM) was employed for classification, regression, and feature selection of multi-class data with homogeneous or heterogeneous features. Lastly, the immensely popular and data-adaptive Random Forest (RF), was utilized to explain correlations and interactions between features, thus making it an ideal integrated tree-based machine learning tool for feature selection.

### 2.6 Functional enrichment analysis of differentially expressed genes

Based on the Gene Ontology (GO) and Kyoto Encyclopedia of Genes and Genomes (KEGG) databases, the pathways affected by DEGs were analyzed through enrichment analysis of DEGs employing the R package “clusterProfiler” (17). The q-value set as the threshold for the adjusted p-value was smaller than 0.05.

### 2.7 Analysis of dynamic expression models (time-series analysis)

Dynamic analysis of gene expression over time, specifically regarding ferroptosis genes in the context of nonalcoholic liver disease in mice and humans, was conducted using the Mfuzz package for fuzzy c-means clustering. The analysis involved six clusters and a fuzzing parameter of 1.25 for all datasets in this study. Subsequently, we identified sets of genes that demonstrated progressively increasing or decreasing expression patterns in both mouse and human samples, and performed enrichment analysis on these sets.

### 2.8 Analysis of ferroptosis patterns in NAFLD

“ConsensusClusterPlus” R package is used for unsupervised consensus clustering taking “module genes” as input data, grounded on k-means machine learning algorithm. This method identified subtypes with varied expression models of ferroptosis molecules. The consensus clustering algorithm was run for 1000 iterations, with 80% of the data samples used in each iteration. The optimal number of clusters was determined using item-consistency plots, the proportion of ambiguous clustering (PAC) algorithms, and by analyzing the relative changes in the area under the cumulative distribution function (CDF) curve. The ferroptosis status of NAFLD patients was assessed by two clusters (namely, “ferroptosis-low” and “ferroptosis-high” groups). To explore the biological significance of this grouping, inter-subgroup difference analysis, enrichment analysis, immuno-infiltration comparison, and comparison of molecular levels of ferroptosis markers were performed.

### 2.9 Immune cell infiltration estimation

The R package “MCPcounter” uses transcriptomic data to precisely quantify the absolute abundance of eight immune cells and two stromal cells (18). We used it to analyze immune cell correlations in NAFLD samples and differences in immune infiltration between ferroptosis subgroups.

### 2.10 Protein–protein interaction

To analyze protein-protein interactions, we generated a PPI network for “module genes” using the STRING database (19). We set a confidence score greater than 0.4 as a critical criterion for selecting relevant gene interactions. Then, we visualized the network using Cytoscape software and identified eight crucial genes in the PPI by applying the Density of Maximum Neighborhood Component (DMNC) method using the CytoHubba plugin (http://hub.iis.sinica.edu.tw/cytohubba/).

### 2.11 Construction of ferroptosis scores by PCA algorithm

To effectively differentiate the two ferroptosis subtypes and evaluate the ferroptosis status of NAFLD patients, we developed a ferroptosis score model using the principal component analysis.

(PCA) algorithm. This model was constructed using the eight essential ferroptosis genes identified through PPI analysis as variables.


Ferroptosis score=∑(PC1+PC2)

The Ferroptosis score was computed by adding up the values of PC1 and PC2, which denote the vital gene expression characteristics of samples in two distinct dimensions. Thus, this score can provide an approximate representation of the ferroptosis pattern.

### 2.12 Single nucleus and single cell analysis

Single-nucleus data were quality controlled and normalized using the Seurat software package, filtering for nFeature_RNA >200 and nFeature_RNA<5000, and percentage of mitochondrial gene expression<5% (percent.mt). All cells within the sample set were downscaled by principal component analysis (PCA) followed by uniform manifold approximation and projection (UMAP). Cell types were globally annotated using the ImmGen and MouseRNAseqData databases from the SingleR software package to ensure accuracy of cell type annotation. We examined the immune profile of eight hub genes by conducting single-cell data analysis of murine liver tissue from GSE158241. Initially, we employed the R package SCTransfom to identify 2000 highly variable genes (n = 2,000). Principal component analysis (PCA) was then conducted, and the JackStraw function was used to select the appropriate principal components for dimensionality reduction. Next, we used the FindClusters function to identify clusters. The resolution was set to 0.4, which generated 16 clusters. Finally, we annotated these clusters based on the expression of known genes and cell types using cell markers and R package xCell.

### 2.13 Western blot

Proteins were isolated from HepG2 cells, and their concentration was determined using a BCA protein assay kit (Sangon Biotech, China). Subsequently, these proteins were separated through a 12% SDS-PAGE gel and transferred onto a nitrocellulose membrane. The membrane was then sealed with 5% bovine serum albumin (BSA) and subsequently incubated overnight at 4°C with the primary anti-ANXA2 antibody (ABclonal, China). The next day, the membranes were subjected to three washes with TBST and subsequently incubated with a horseradish peroxidase-labeled secondary antibody (1:4000) for 1 hour at room temperature. Color development was achieved by adding BeyoECL Moon (Beyotime Biotechnology, China). GAPDH was sourced from Sangon Biotech.

### 2.14 Cell culture and siRNA transfection

Human hepatoma cell line HepG2(American Type Culture Collection, USA) maintained in DMEM medium encompassing 10% fetal bovine serum (Gibco, Grand Island, USA) and 1% penicillin-streptomycin (Gibco, Grand Island, USA) and cultured at 37°C and 5%CO2. ANXA2-siRNA was obtained from Sangon Biotechnology and used to silence ANXA2 expression. Before transfection, HepG2 cells were seeded in 6-or 12-well plates and grown to 60% to 70% of the cell confluence for transfection. ANXA2 siRNA/NC siRNA was transfected into HepG2 cells for 48 h at a final concentration of 10nM using siRNA transfection reagent RNATransMate(Sangon, Shanghai, #E607402). Successfully transfected cells were used in subsequent experiments. In this study, the sequence of ANXA2 siRNA was as follows: siANXA2-1 5 ‘-TGAGGGTGACGTTAGCATTAC-3’; siANXA2-2 5 ‘-CGMGGATGCTTTGAACATTGAA-3’. The NC siRNA sequence was as follows: 5 ‘-UUCUCCGAACGUGUCACGUTT-3’.

### 2.15 Determination of ferrous ion content and its localization in mitochondria

After the cells were grouped and treated in different ways, the medium was removed. After washing with PBS, serum-free medium containing FerroOrange (Dojindo, Japan) and Mitotracker Green (Meiunbio) was added, respectively. The cells were incubated in a 5% CO2 incubator at 37°C for 30 min and finally observed under a fluorescence confocal microscope (FV3000, Olympus, Japan).

### 2.16 Determination of lipid peroxidation

After the cells were grouped and treated in different ways, the medium was removed and C11 BODIPY 581/591(Invitrogen, D3861) was added. After incubation at 37°C for 1 h in a 5% CO2 incubator, the plates were washed twice with PBS to remove excess dye. Cells were digested with trypsin and then re-suspended in 5% PBS for flow cytometry (Cytek, USA) analysis.

### 2.17 Reactive oxygen species analysis

The Reactive Oxygen Species Assay Kit (Beyotime, Shanghai, China) is a kit that uses a DCFH-DA fluorescent probe to detect ROS. The cells were seeded in 12-well plates, and the cells were grouped in different ways. After dilution of DCFH-DA(1:1000) with serum-free 1640 medium in the dark, 500μL per well was added to a 12-well plate and incubated at 37°C in a 5% CO2 incubator for 30 min. After washing with PBS, the cells were trypsinized and transferred to flow tubes, and reactive oxygen species levels in the cells were analyzed using flow cytometry (Cytek, USA) or fluorescence confocal microscope (FV3000, Olympus, Japan).

### 2.18 Measurement of superoxide anion levels

Dihydroethidium(DHE) is the most commonly used fluorescent probe for detecting intracellular superoxide anion levels, which can react with intracellular superoxide anion to produce Ethidium. Ethidium binds to RNA or DNA to produce red fluorescence. Therefore, the higher the level of superoxide anion in cells treated with DHE, the stronger the red fluorescence, and the changes in its level can be analyzed by flow cytometry and fluorescence confocal microscope. In this experiment, DHE and cells were co-incubated at 37°C, 5% CO2 incubator for 30 min for fluorescent probe loading, followed by PBS washing, and finally detected and analyzed by flow cytometry(Cytek, USA) and fluorescence confocal microscope(FV3000, Olympus, Japan).

### 2.19 Statistical analysis

The statistical analysis of bioinformatics were performed using R software (https://www.r-project.org/, version 4.2.1). The analytical and statistical software for the experiments was ImageJ and Graphpad prism.

## 3 Result

### 3.1 Weighted gene co-expression network construction

**Figure 1:**
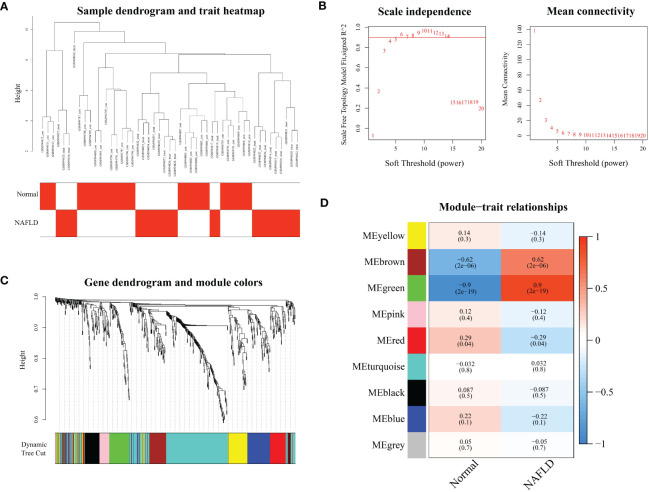
Modular analysis of weighted gene co-expression network analysis. **(A)** Sample clustering dendrogram for each sample corresponding to the leaves of the tree is cut at 10.5. **(B)** Analysis of the scale-free index and the mean connectivity for various soft-threshold powers. **(C)** Displays the merged modules under the cluster tree. **(D)** The correlations and corresponding p-values of each cell are presented about Module-trait correlations.

The expression matrix of 659 ferroptosis genes in GSE40481 was used as input data for WGCNA. By setting the threshold, two samples with significant abnormalities were excluded (**Figure 1A**). The optimal soft threshold power was identified as 4 with high average connectivity when R^2 = ^0.9, as shown in **Figure 1B**. Similar modules were combined, resulting in the identification of nine modules, as shown in **Figure 1C**. The brown and green modules were both positively correlated with NAFLD: the brown module had a correlation of 0.9 (p=2e-19), while the green module had a correlation of 0.62 (p=2e-6), as displayed in **Figure 1D**. These two clinically significant modules, which contain a total of 135 ferroptosis genes, were further utilized for genetic screening.

### 3.2 Identification of aberrantly expressed ferroptosis genes in NAFLD

**Figure 2:**
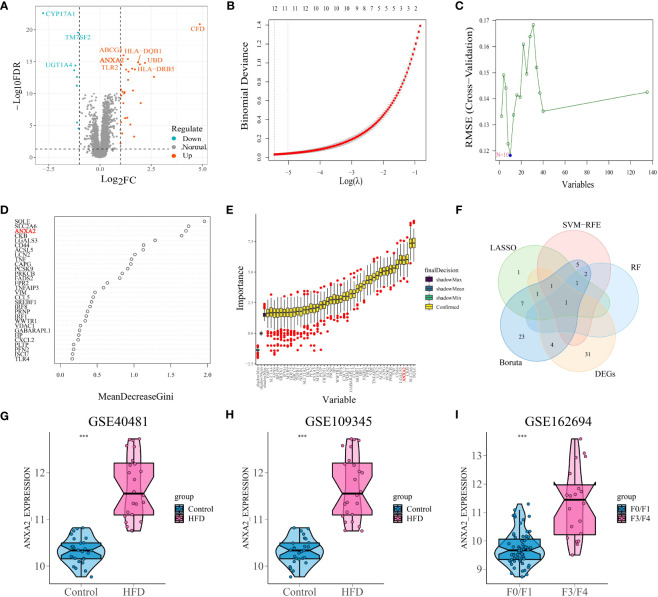
Differential analysis and selection of candidate characteristic biomarkers in module genes based on machine learning algorithms. **(A)** Volcano plots displayed the differentially expressed gene with the criteria of |logFC| > 1 and p-value< 0.05. The red and green circles indicate the up-regulated and down-regulated DEGs, respectively. **(B)** By conducting cross-validation to select the optimal tuning parameter log (Lambda) in LASSO regression analysis, 12 genes were ultimately obtained. **(C)** 10 genes obtained using the SVM algorithm. **(D)** The top 4 genes with MeanDecreaseGini > 1.5 were selected. The rank of genes is shown according to their relative importance. **(E)** With importance ranking, 37 selected typical genes by Boruta. **(F)** Venn diagram shows that the candidate characteristic gene ANXA2 is identified via the above 4 machine learning algorithms and differential analysis. **(G, H)** The expression of ANXA2 in GSE40481 and GSE109345, respectively. “***” means that p < 0.001. **(I)** Differential expression of ANXA2 in mild versus severe fibrosis. “***” means that p < 0.001.

Analyses were performed using differential analysis to identify genes exhibiting aberrant expression in NAFLD. Ultimately, researchers identified 29 up-regulated genes and 9 down-regulated genes, which are compiled in **Supplementary Table S2**. A graphical representation of the differentially expressed genes is depicted using a volcano plot (**Figure 2A**).

### 3.3 Machine learning algorithms identify the target gene ANXA2

The combined results of fold change and four machine learning algorithms localized the core genes of the study. By utilizing LASSO regression to streamline the ferroptosis module genes, 12 potential markers that may serve as useful indicators for NAFLD were uncovered (**Figure 2B**). SVM-RFE method selected 10 genes as important biomarkers (**Figure 2C**). Randomforest algorithm listed the importance score for each gene, with ANXA2 ranked third (**Figure 2D**). And the fourth place in the ranking appears in Boruta (**Figure 2E**). Five screening methods finally yielded ANXA2, the core gene of this study (**Figure 2F**; **Supplementary Table S3**). **Figures 2G–I** validated the robustness of the machine learning screening results. The expression of ANXA2 was significantly elevated in both mouse datasets and one human dataset, while the ROC curves of single genes demonstrated satisfactory AUC values (0.992, 0.953 and 0.892, respectively) (**Supplementary Figure S2**). Therefore, ANXA2 was selected as the core gene for subsequent analysis.

### 3.4 Functional enrichment analysis of differentially expressed genes

To investigate the pathways affected by DEGs, we conducted GO and KEGG analyses. Regarding the three levels of GO analysis biological process (BP), cellular component (CC), and molecular (MF) (**Supplementary Figure S3A**), we observed a high enrichment of DEGs in various pathways such as “antigen processing and presentation”, “MHC class II protein complex assembly”, “integral component of lumenal side of endoplasmic reticulum”, “lumenal side of endoplasmic reticulum membrane”, “lumenal side of endoplasmic reticulum membrane “, “MHC class II protein complex binding”, “MHC protein complex-binding immunoreceptor activity” and “Immunoglobulin binding”. Furthermore, the KEGG enrichment analysis revealed several immune-related signaling pathways (**Supplementary Figure S3B**), including “antigen processing and presentation” and “Th cell differentiation “, among others. The reason we obtained such enrichment results is due to the presence of multiple genes encoding human leukocyte antigens (HLA) in the differential genes. Several studies have found that polymorphisms of HLA molecules are associated with susceptibility to NAFLD and grading of lesions (20). Also, HLA molecules have been associated with inflammatory response and immune function in NAFLD (21). This suggests that the relationship between the immunity and NAFLD needs to be further studied and explored.

### 3.5 Expression pattern analysis of FRGs

**Figure 3:**
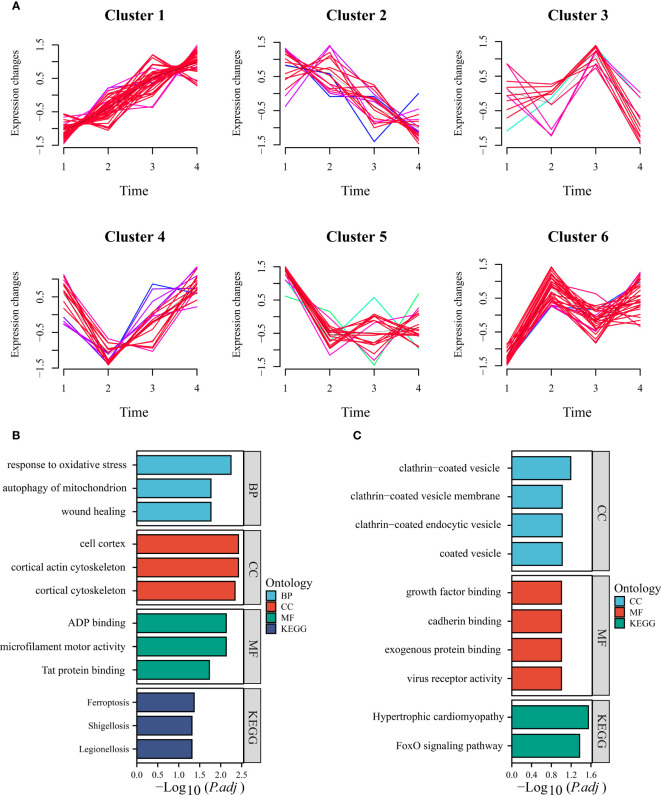
Time series analysis of module genes. **(A)** Time-based analysis facilitated the derivation of six clusters (clusters 1, 2, 3, 4, 5, and 6). **(B)** The bar chart presented herein showcases the up-regulation of GO and KEGG terms characteristic of cluster 6. **(C)** Conversely, the bar chart provided depicts the consistency of down-regulated GO and KEGG terms exhibited by cluster 3.

The development of NAFLD progresses through multiple stages, making it crucial to analyze gene expression dynamics. Using the training set, we clustered the “module genes” based on their temporal expression patterns, yielding six clusters. Cluster 1 comprised genes that were progressively up-regulated during high-fat diet induction, while cluster 2 showed a reverse trend (**Figure 3A**). Enrichment analysis of genes from these two clusters showed that they are associated with biological pathways such as oxidative stress and ferroptosis (**Figures 3B, C**). Notably, ANXA2 is classified into progressively upregulated gene group, suggesting its potential to describe disease progression. In the additional mouse dataset, we obtained consistent results that supported our idea (**Supplementary Figure S4A**). Moreover, ANXA2 manifested in a cluster positively correlated with the stage of liver fibrosis (F0-F4) in our human dataset, indicating its importance in the progression of NAFLD, especially during fibrosis (**Supplementary Figure S4B**). By intersecting the upregulated gene sets, we identified 23 ferroptosis genes that merit further exploration (**Supplementary Table S4**).

### 3.6 Consensus clustering approach to develop ferroptosis subtypes in NAFLD

**Figure 4:**
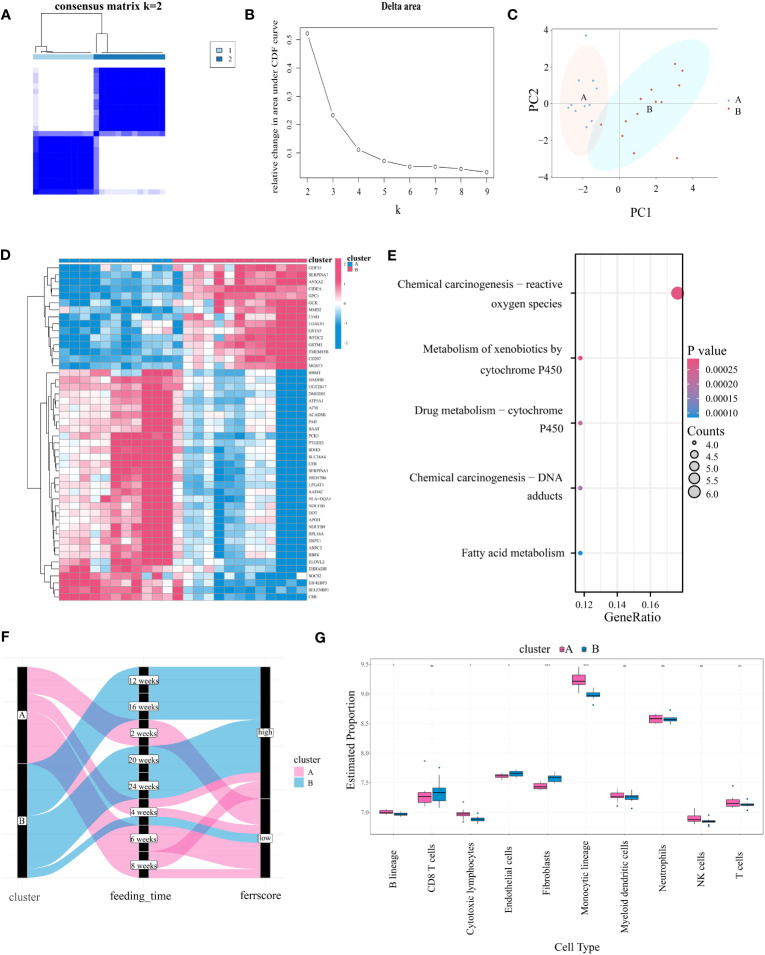
Identifying Ferroptosis Subgroups Through Unsupervised Consensus Clustering Algorithms. **(A)** Two ferroptosis subgroups were ascertained the optimal value for consensus clustering. **(B)** The corresponding relative area changes under the cumulative distribution function (CDF) curve. k takes values ranging from 2 to 9, with the optimal k = 2. **(C)** The PCA plot shows two divided ferroptosis subclusters. **(D)** The heatmap showing differential gene expression between the two ferroptosis isoforms. **(E)** KEGG analysis of differentially expressed gene in two subgroups. **(F)** Sankey diagram between ferroptosis isoforms, high-fat diet feeding time and ferroptosis score (with cutoff value at 0, greater than zero for high score and less than zero for low score). **(G)** Differences in immune cell expression between ferroptosis subgroups by MCPcounter. "*” means that p < 0.05; “**” means that p < 0.01; “***” means that p < 0.001; ns, no significance.

We utilized the consensus clustering method on the gene expression data of the “module genes” from 24 high-fat diet-induced mouse samples of GSE40481. This approach identified two ferroptosis subtypes (A:B=11:13) with highly consistent gene expression patterns within subtypes (**Figures 4A, B**), which were clearly separated in the PCA plot (**Figure 4C**). Genes that were clearly differentially expressed between subtypes were shown by heatmap (**Figure 4D**). Differential genes were mainly enriched in “chemical carcinogenesis-reactive oxygen species”, “cytochrome P450 metabolism of allosteric substances”, “fatty acid metabolism” and other pathways (**Figure 4E**). Additionally, we found that the subtypes were closely associated with the duration of high-fat diet feeding. Specifically, cluster B was linked to more prolonged feeding, indicating a more severe degree of the disease, whereas cluster A represented a milder degree (**Figure 4F**). The same finding was also seen in GSE109345 (**Supplementary Figure S5A**). In the human dataset, the “module genes” similarly divided the samples into two categories - cluster A and cluster B. Meanwhile, we were surprised to find that the former one included almost all patients with lower fibrosis stages and the latter cluster shows the opposite (**Supplementary Figure S5B**). In summary of the results, we conclude that ferroptosis typing is likely to be closely associated with the degree of steatosis in mice and fibrosis in humans, which at once emphasizes that FRGs may drive and characterize the course of NAFLD disease, and, this typing scheme is promising.

### 3.7 Evaluation and analysis of immune cell infiltration

In order to explore the correlation between the expression levels of FRGs and immune cells, an immune infiltration analysis was conducted through utilization of the MCPcounter algorithm. The immune cell correlation of the disease groups is shown in **Supplementary Figure S6B**, notably Fibroblasts showed a positive correlation with Endothelial cells (R=0.79) and a negative correlation with Monocytic lineage (R=-0.42). In addition, the correlation between T cells and CD8 T cells was 0.67. **Figure 4G** shows the difference in immune cell infiltration between ferroptosis subtypes. Compared to cluster A, “Endothelial cells” and “ Fibroblasts” were significantly upregulated in cluster B, while “B lineage”, “Cytotoxic lymphocytes” and “Monocytic lineage” were significantly downregulated. We also analyzed four immune checkpoints between subtypes. Results showed that CD274 (encoding programmed death ligand 1) was highly expressed in cluster A, while HAVCR2 (encoding hepatitis A Virus Cellular Receptor 2) was highly expressed in cluster B (**Supplementary Figure S6A**). Our analysis also revealed significant differences in the levels of ferroptosis markers between subtypes, with ACSL4, HIF1A, MAPK1, GPX4, ISCU, CAV1 and SLC7A1 significantly upregulated in subtype B, suggesting that subtype B has a more severe degree of ferroptosis, which is likely to be associated with the course of NAFLD (**Supplementary Figure S6C**).

### 3.8 Ferroptosis score construction

**Figure 5:**
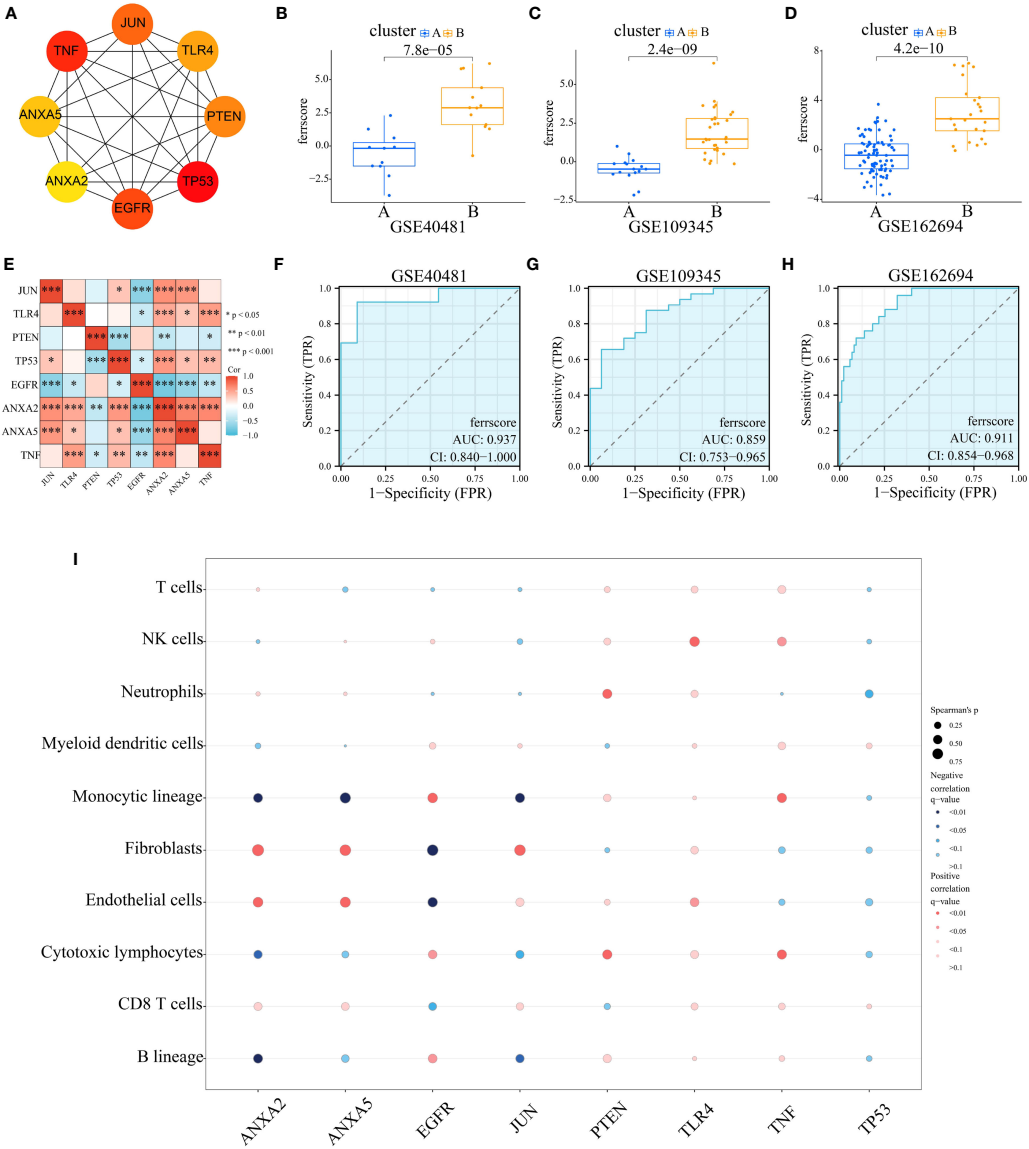
Construction and Validation of Ferroptosis Score. **(A)** Using Cytoscape and CytoHubba to identify 8 modular genes to construct ferroptosis score and visualize molecular interaction networks. **(B–D)** Differences in ferroptosis score between two ferroptosis subgroups in GSE40481, GSE109345 and GSE162694. **(E)** Correlation between ferroptosis score genes. **(F–H)** The receiver operating characteristic (ROC) curve for differentiating ferroptosis subgroups by ferroptosis score in GSE40481 (AUC=0.937), GSE109345 (AUC=0.859) and GSE162694 (AUC=0.911). **(I)** Bubble diagram of the relationship between 8 score genes and immune cells.

To differentiate subtypes and quantify the level of ferroptosis, we constructed an ferroptosis score. Cytohhuba plugin of the DMNC algorithm identified eight key genes, including JUN, TLR4, PTEN, TP53, EGFR, ANXA2, ANXA5, and TNF (**Figure 5A**). Correlation analysis revealed statistically significant associations between ANXA2 and all other seven genes (**Figure 5E**). Subsequently, these eight genes were incorporated into the development of the ferroptosis score. Principal components 1 and 2 were chosen, and the score was determined through implementation of principal component analysis (PCA). Ferroptosis score varied significantly among subtypes, with subtype 2 receiving higher score in all three data sets (**Figures 5B–D**). High AUC values were shown in **Figures 5F–H** (0.937, 0.859, and 0.911, respectively), indicating that the subtype classification efficacy of the score was reliable. Additionally, an exploration was conducted regarding the correlation between the scored genes and immune cells, as is demonstrated in **Figure 5I**. The analysis indicated that ANXA2 exhibited a significant positive correlation with “Fibroblasts”, as well as a negative correlation with “Monocytic lineage” and “Endothelial cells”. **Supplementary Figures S7A–F** provides further clarification on these findings. ANXA5 displayed similar results. However, EGFR showed opposite results from ANXA2 and ANXA5. This may be because EGFR belongs to the genes progressively downregulated during disease progression, whereas ANXA2 and ANXA5 belong to the progressively upregulated one. The above results led us to speculate that “fibroblasts”, “monocyte lines” and “endothelial cells”, which may undergo some regular changes during the progression of the disease, may be associated with the expression of FRGs.

### 3.9 Single-nucleus RNA-seq and single-cell RNA-seq analysis

Single-nucleus analysis showed that hepatocytes were the predominant cells in the samples in both the normal and disease groups, and our target gene ANXA2 was significantly more expressed in NAFLD hepatocytes compared to the normal group (**Supplementary Figures S8A–D**). To further examine the correlation between genes responsible for scoring and immune cells in NAFLD, we scrutinized scRNA-seq data extracted from liver biopsy samples of high-fat diet-driven NASH mice and their corresponding control samples. With the aid of xCell, we annotated ten distinct cell types (**Supplementary Figure S8E**). The outcome depicted ANXA2 and ANXA5 to be predominantly expressed by monocytes, macrophages, and epithelial cells (**Supplementary Figure S8F**). This finding hints towards the pivotal involvement of monocytes, macrophages, and epithelial cells in the development of NAFLD.

### 3.10 Silencing ANXA2 gene alleviated erastin-induced ferroptosis in HepG2 cells

**Figure 6:**
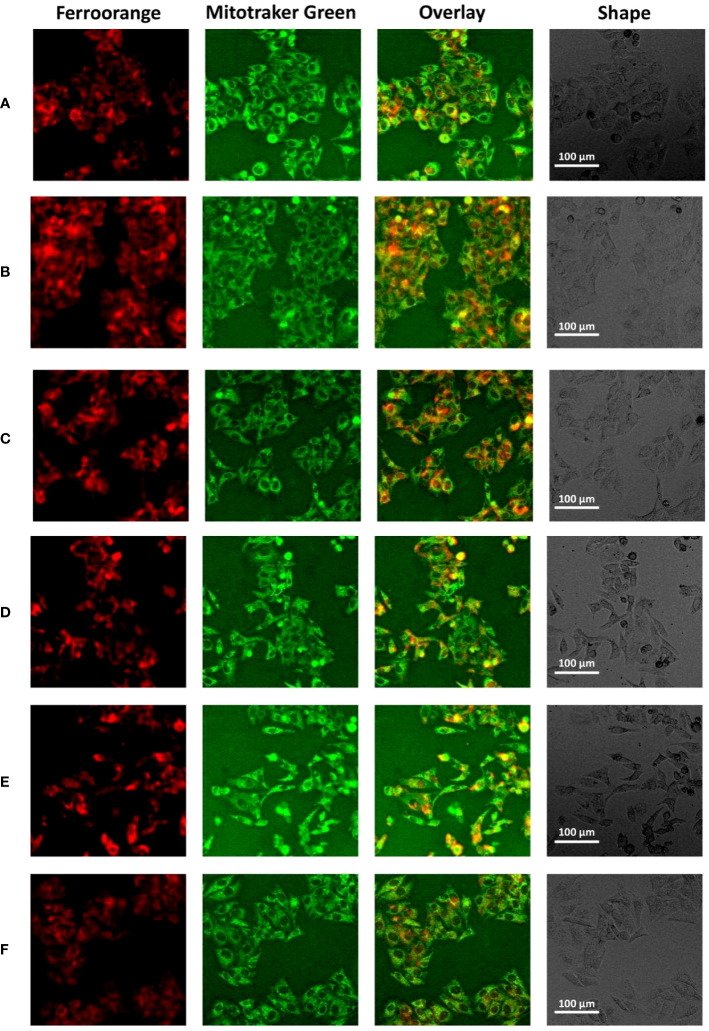
The effect of ANXA2 on Fe2+ levels in cells. **(A)** Normal control HepG2 cells; **(B)** HepG2 cells treated erastin (20 μM) for 24 h; **(C)** HepG2 cells co-cultured with siRNA control for 48 h and then treated erastin (20 μM) for 24 h; **(D)** HepG2 cells co-cultured with siANXA2-1 control for 48 h and then treated erastin (20 μM) for 24 h; **(E)** HepG2 cells co-cultured with siANXA2-2 control for 48 h and then treated erastin (20 μM) for 24 h; **(F)** HepG2 cells treated erastin (20 μM) and Ferrostatin-1 (2 μM) for 24 h. (The scale bar indicates 100 μm length, cells were incubated with Ferroorange (red color, Texas Red channel) and Mitotracker Green (green color, FITC channel) for 30 min to label intracellular Fe2+ and mitochondria, respectively).

We used HepG2 cells to verify the relationship between the core gene ANXA2 and ferroptosis *in vitro*. HepG2 cells were transfected with ANXA2 siRNA for 48 hours, and the cells were stimulated with ferroptosis inducer erastin. **Supplementary Figures S9A,B** shows that ANXA2 was successfully knocked down. In addition, the intracellular ferrous ion level and mitochondrial changes were observed. As shown in **Figure 6** and **Supplementary Figure S9C**, compared with the control group, the intracellular ferrous ion concentration was significantly increased, the fluorescence intensity of mitochondria was decreased, and the co-localization of mitochondria and iron ions was obvious after stimulation of cells with erastin alone (**Figures 6A, B**). There were no significant changes in the intracellular ferrous ion level and mitochondria when the siRNA control was used (**Figure 6C**). However, after the silencing of ANXA2 in HepG2 cells mediated by two siRNAs, the fluorescence intensity of ferrous ions decreased, the fluorescence intensity of mitochondria increased, and the colocalization decreased (**Figure 6D, E**), which was consistent with the changes after the intervention of ferroptosis inhibitor Ferrostatin-1 in the positive control (**Figure 6F**). These results indicated that the deposition of ferrous ions in HepG2 cells, especially in mitochondria, was alleviated after inhibiting the ANXA2 gene.

**Figure 7:**
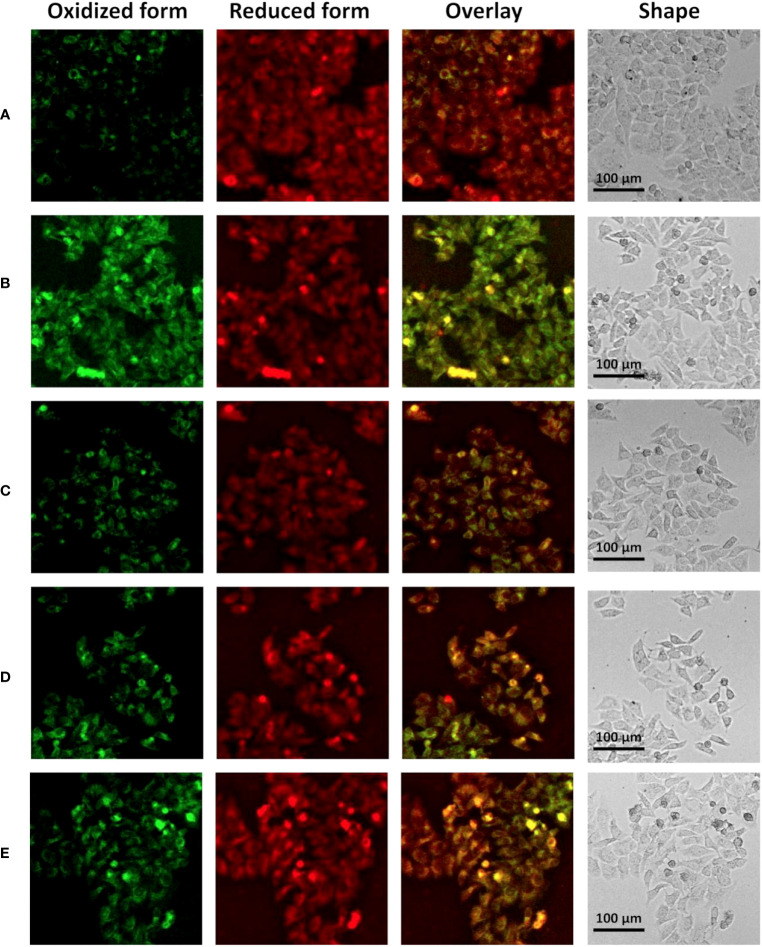
The effect of ANXA2 on lipid peroxidation in cells. **(A)** Normal control HepG2 cells; **(B)** HepG2 cells co-cultured with siRNA control for 48 h and then treated erastin (20 μM) for 24 h; **(C)** HepG2 cells co-cultured with siANXA2-1 control for 48 h and then treated erastin (20 μM) for 24 h; **(D)** HepG2 cells co-cultured with siANXA2-2 control for 48 h and then treated erastin (20 μM) for 24 h; **(E)** HepG2 cells treated erastin (20 μM) and Ferrostatin-1 (2 μM) for 24 h. (The scale bar indicates 100 μm length, cells were incubated with C11 BODIPY 581/591 for 60 min to label lipid peroxide, the FITC channel (green color) indicates oxidized form while the Cy5 channel (red color) indicates the reduced form).

**Figure 8:**
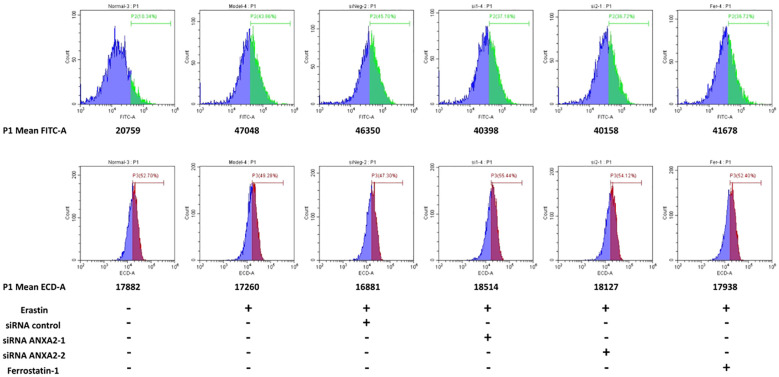
The effect of ANXA2 on ROS and DHE levels in cells. HepG2 cells were co-cultured with siRNA for 48 h and then treated erastin (20 μM) for 24 h; Ferrostatin-1 (2 μM) were co-treated for 24 h. (DCFH-DA and DHE for 30 min to label intracellular ROS and superoxide anion, respectively).

When ferroptosis occurs, intracellular lipid peroxidation and reactive oxygen species (ROS) are often increased. Consistently, we further examined important intracellular oxidative metabolites. It was found that compared with the control group, intracellular lipid peroxidation was significantly increased (enhanced green fluorescence) after treatment with erastin and siRNA control (**Figures 7A, B**), while lipid peroxidation was significantly alleviated after silencing ANXA2 gene (**Figures 7C–E**; **Supplementary Figure S9D**). In addition, we used DCFH-DA and DHE to characterize intracellular ROS and superoxide anion levels. Flow cytometry analysis showed that ROS level was significantly up-regulated after erastin stimulation (average fluorescence intensity 47048, average fluorescence intensity of the normal control group was 20759) (**Figure 8**). There was little change after siRNA control treatment, while the average fluorescence intensity of ROS decreased to about 40000 after ANXA2 inhibition, and to 41678 after intervention with the positive control Ferrostatin-1 (**Figure 8**). DHE did not change significantly between groups. The trend of ROS and DHE under fluorescence confocal microscope was consistent with the results of flow cytometry (**Supplementary Figure S10**). The above data proved that the intracellular oxidative stress was significantly relieved after ANXA2 inhibition. Based on the above data, we demonstrated that ANXA2 gene inhibition could alleviate erastin-induced ferroptosis in HepG2 cells.

## 4 Discussion

NAFLD has been shown to be associated with ferroptosis in previous reports, however, we still lack evidence at the molecular level. The aim of this study was to analyze the role of ferroptosis molecules in NAFLD based on high-throughput data. First, we used WGCNA combined with four machine learning methods to identify the most robust core gene, ANXA2. We observed a consistent upregulation of ANXA2 during the course of the disease through time-series analysis. Furthermore, we developed a ferroptosis typing scheme for NAFLD using an unsupervised consensus clustering approach. The significant differences between two subtypes in terms of disease course, immune cells level, and ferroptosis marker molecule level provide promise to improve the existing subtype classification. Finally, we conducted *in vitro* experiments using HepG2 cells and found that ANXA2 may play a role in promoting ferroptosis, which has not been previously reported.

To our knowledge, the identification of targets for NAFLD treatment using bioinformatics approaches is uncommon so far. Only a few results have provided the following targets, including ENO3, CXCL10, INHBE, LRRC31, OPTN (22) and IGFBP-2 identified by Wen et al. (18). This is far from sufficient for targeted therapy of NAFLD. ANXA2 is a new target we have identified, which is a phosphatidylinositol binding protein located on the surface of exosomes. With the available evidence, ANXA2 expression levels have been found to be significantly elevated in patients with NAFLD, while its deletion can prevent the development of liver injury (23). Notably, ANXA2 has multiple roles in the pathophysiological process of NAFLD. First, ANXA2 is lowly expressed in normal liver tissues, whereas abundantly expressed in acute liver injury, due to its ability to bind to tissue-type fibrinogen activator to promote hepatic neovascularization and its repair (24, 25). Secondly, ANXA2 is involved in regulating the p-STAT3/ANXA2 axis, which induces hepatocyte pyroptosis (23). Not only that, ANXA2 may also promote hepatic stellate cell activation and collagen fibril synthesis by participating in the Anxa6/miR-9-5p/Anxa2 pathway or increasing the expression of osteopontin, thus leading to the onset and acceleration of hepatic fibrosis (26, 27). And our experimental results firstly reveal that ANXA2 may induce ferroptosis, thereby further exacerbating liver injury.

The proposal that ferroptosis is involved in the pathogenesis and progression of NAFLD only emerged after 2019. One of the initial studies indicated that while both apoptosis and necrosis of hepatocytes may contribute to the development of nonalcoholic steatohepatitis (NASH), ferroptosis is the primary mode of cell death during the transition from simple steatosis to steatohepatitis. The study’s experimental results demonstrated that the inhibition of necroptosis alone did not prevent cell death onset, whereas the inhibition of ferroptosis almost entirely protected hepatocytes from death while suppressing the subsequent infiltration of immune cells and inflammatory response (28). Another early study revealed that GPX4 and its associated ferroptosis promoted NASH induced through methionine/choline-deficient diet (MCD) feeding (29). Subsequently, mechanisms related to ferroptosis’s involvement in NAFLD were clarified gradually, with molecular-level evidence including reduced GPX4 activity, upregulation of ACSL4 (due to arsenic induction) and the suppression of Nrf2 pathway, together with iron overload and lipid peroxidation (30). Based on these mechanisms, several approaches that target the ferroptosis pathway for treating NAFLD have been explored, including sodium selenite (SS) (29), thymosin β4 (Tβ4) (31), and ENO3 (32), which modulate GPX4 to inhibit ferroptosis. Iron removal therapy has been shown to reduce alanine aminotransferase levels in hepatocytes (33). Rosiglitazone (ROSI), an ACSL4 inhibitor, can also suppress arsenic-induced ferroptosis (34). Additionally, there are recognized inhibitors of ferroptosis, including Fer-1 (35), LPT-1, and DFP (29), which possess definite mitigatory effects on NAFLD. But in general, these leads are incomplete, particularly in establishing the molecular targets of ferroptosis implicated in each stage of NAFLD.

ANXA2 is filtered by machine learning algorithms. A previous study of ours also demonstrated the robustness of the machine learning results (36). In this study, the differential expression of ANXA2 in the three datasets represents its reliability. Furthermore, our study reveals for the first time that ANXA2 is closely associated with disease course while being differentially expressed in NAFLD. ANXA2 expression gradually increased with increasing feeding time on a high-fat diet in mouse samples, and in human samples, its expression was also upregulated with the severity of liver fibrosis stage. This suggests that ANXA2 may act in all stages of NAFLD, and that this action is negative. It may promote the development of NAFLD by regulating lipid metabolism, activating the inflammatory response (37) and influencing the fibrotic process (23). There are another 22 genes with the same expression trend, and these deserve to be further investigated. Although there are no reports on the association of ANXA2 with ferroptosis, the results of our experiments suggest that it may act as a target to rescue it by reducing the accumulation of iron, lipid peroxidation, reactive oxygen species (ROS) levels, and superoxide anion levels in cells. It is worth mentioning that ANXA2 regulates ROS in previous studies more as a protective factor for down-regulation of ROS. He et al. found that ANXA2 showed a negative correlation with ROS levels in sepsis models (38), which is different from our experimental results. One possible explanation for this discrepancy is that ANXA2 promotes ferroptosis by causing upregulation of ROS levels through the inverse regulation of PRDX2 levels (39). Two recent studies have provided evidence that PRDX2 can inhibit cellular ferroptosis (40, 41). Therefore, we suggest that ANXA2 may have a dual role in the regulation of redox, and its downregulation of antioxidant molecular activity may be important in promoting the occurrence of ferroptosis. Additionally, the mechanism of how the expression profile of ANXA2 affects ferrous ion levels in mitochondria remains unclear and requires further analysis.

Our study provides a typing scheme for NAFLD based on FRGs. It is significant that it can well differentiate different groups of samples in the dataset. JUN, TLR4, PTEN, TP53, EGFR, ANXA2, ANXA5, and TNF were the genes we screened for scoring, and correlation analysis showed that ANXA2 was associated with all of these genes. Notably, JUN, TLR4, and ANXA5, like ANXA2, showed a trend of upregulation with increasing NAFLD in our three datasets. JUN, TLR4 (42), EGFR (43), and TNF (44) were confirmed to promote the progression of NAFLD in previous studies, while ANXA5 (45) and PTEN (46) were identified as protective factors. TP53 has a dual role in NAFLD (47). TLR4 antagonist Sparstolonin B (SsnB) inhibited TLR4-induced liver fibrosis. The mechanism may include upregulation of PTEN protein expression to reduce TLR4-PI3k akt signaling and increased p53 gene and protein expression. In addition, SsnB may also reduce fibrosis by antagonizing TLR4-induced TGFβ signaling pathway (48). Another analysis manifests that SsnB may exert an anti-NASH effect by reducing the transport of TLR4 to lipid rafts (49). Not only that, a recent experimental study demonstrated that Zeaxanthin (ZEA) has the ability to effectively decrease the expression of p53, in turn regulating downstream targets such as GPX4, SLC7A11, SAT1, and ALOX15. These actions cumulatively contribute to the effective inhibition of ferroptosis in NAFLD cells (50). In summary, the inter-regulation between these FRGs on which our typing scheme is based plays an important role in NAFLD and they deserve further investigation. More evidence is needed to target them to inhibit ferroptosis occurrence and thus treat NAFLD.

Our study provides evidence supporting the involvement of ferroptosis molecules in NAFLD. However, there are some limitations to our findings. Firstly, the duration of the high-fat diet may not fully reflect the severity of NAFLD, which could have influenced the positive results obtained. In addition, although we demonstrated that knockdown of ANXA2 in the presence of iron metastasis inducers inhibited ROS levels, more experimental studies are still needed to investigate how ANXA2 regulates ROS levels. However, our findings indicate that ANXA2 may possess a high degree of potential as a target for the regulation of the ferroptosis pathway in individuals affected by NAFLD.

## 5 Conclusion

Our bioinformatics analysis revealed ANXA2 as a key gene in NAFLD pathogenesis with diagnostic potential. ANXA2 expression was found to be positively associated with the course of NAFLD and increased along with liver fibrosis in human samples. Additionally, we propose a new typing scheme for NAFLD based on FRGs expression. Experimental results suggest that ANXA2 is an important target in suppressing ferroptosis and could potentially aid in NAFLD treatment.

## Data availability statement

The original contributions presented in the study are included in the article/**Supplementary Material**. Further inquiries can be directed to the corresponding author.

## Author contributions

JQ: Investigation, Methodology, Visualization, Writing – original draft. PC: Data curation, Resources, Validation, Writing – review & editing. XD: Data curation, Formal Analysis, Investigation, Software, Writing – original draft. ZZ: Investigation, Validation, Writing – original draft. LD: Writing – original draft. LL: Conceptualization, Funding acquisition, Project administration, Supervision, Writing – original draft, Writing – review & editing.
