# Supplementary material for: Machine learning identifies ferroptosis-related gene ANXA2 as potential diagnostic biomarkers for NAFLD
Source: Front Endocrinol (Lausanne). 2023 Dec 19;14:1303426. doi: 10.3389/fendo.2023.1303426 (PMC10773757; doi:10.3389/fendo.2023.1303426)
Supplement: Supplementary file 1 [file DataSheet_1.docx]

Supplementary Material

**Machine learning identifies ferroptosis-related gene ANXA2 as potential diagnostic biomarkers for NAFLD**

**Jingtong Qin^1†^，Peng Cao^2†^，Xuexuan Ding^1^，Zeyao Zeng^1^，Liyan Deng^1^, Lianxiang Luo^3,4*^**

***Correspondence:** luolianxiang321@163.com


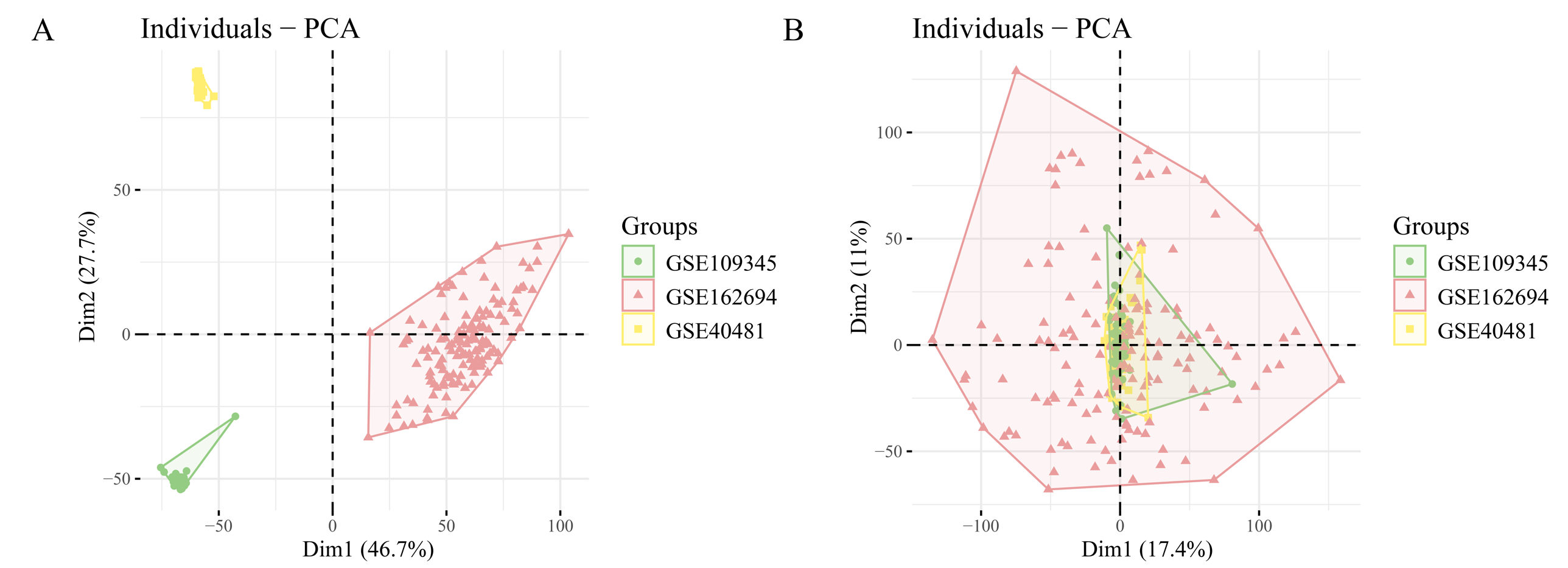


**Supplementary Figure 1**. Principal Component Analysis (PCA). The samples based on the top two principal components (PC1 and PC2) of gene expression profiles before**(A)**and after**(B)** batch effect.


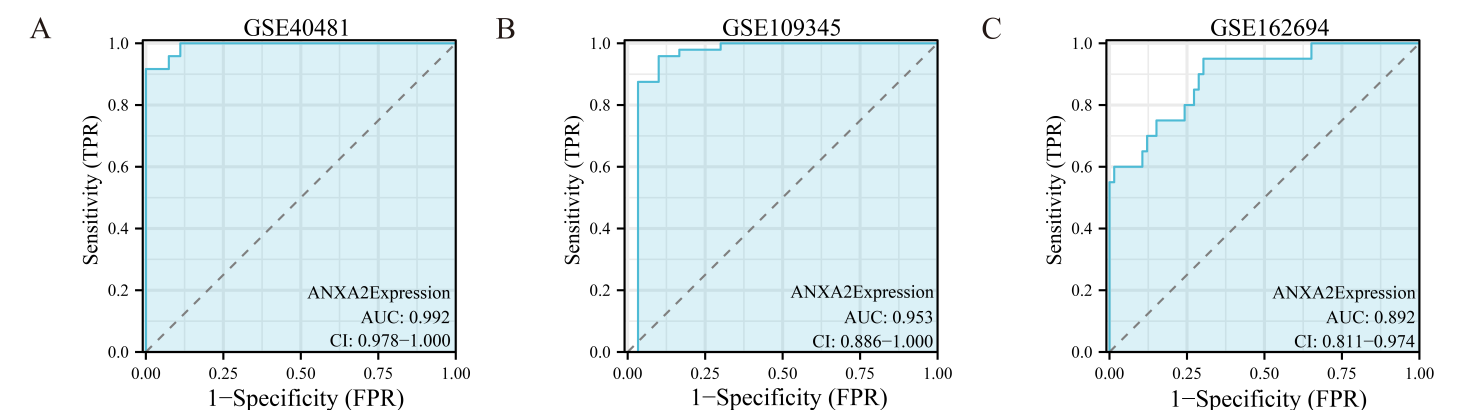


**Supplementary Figure 2**. Validation of ANXA2 Expression to Distinguish the Normal Group from NAFLD. **(A-C)** The receiver operating characteristic (ROC) curve of ANXA2 gene diagnosis of NAFLD in GSE40481, GSE109345 and GSE162694.





**Supplementary Figure 3**. Functional enrichment analysis of differentially expressed genes. **(A)** Gene ontology enrichment analysis of DEGs encompassing molecular function, cellular component, and biological process. **(B)** KEGG enrichment analysis of DEGs.





**Supplementary Figure 4**. Mfuzz Cluster Analysis of “Module genes” in GSE109345 and GSE162694. **(A)** The expression of "module genes" changes on a time scale of high-fat diet feeding for 0week, 6week, 12week, 18week and 24week in GSE109345. **(B)** The expression of "module genes" changes with fibrosis stage 0, 1, 2, 3, and 4 in GSE162694.


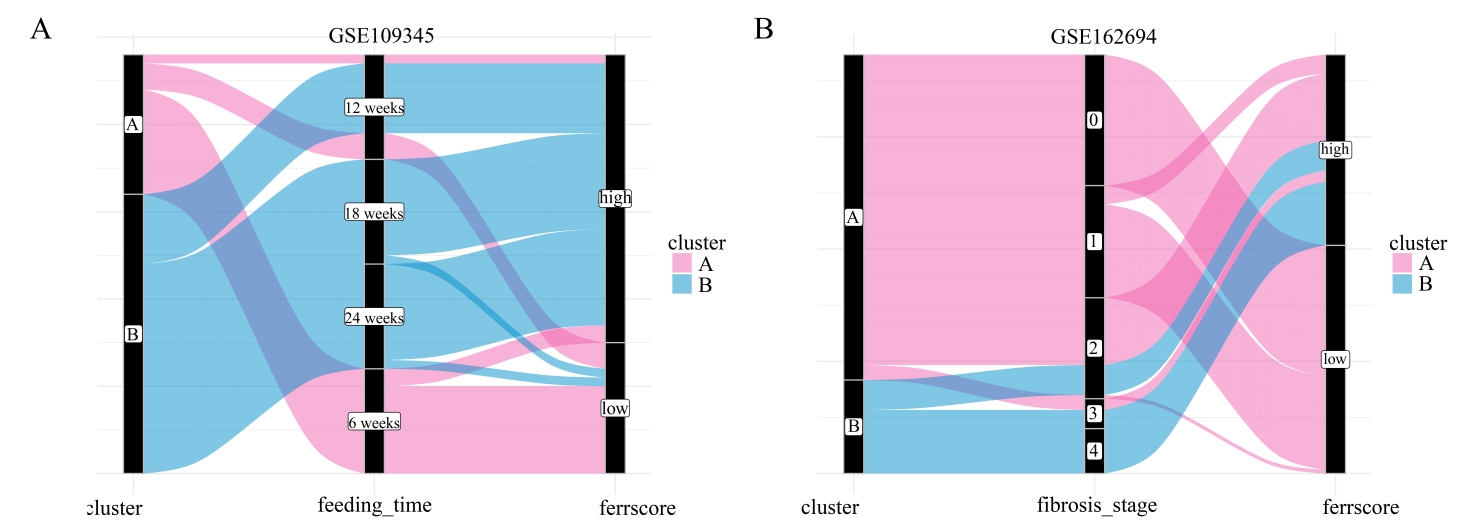


**Supplementary Figure 5**. Sankey Plots of Ferroptosis subtypes, high-low ferroptosis score groups (with a scoring threshold of 0), and experimental/clinical variables. **(A)** The relationship between ferroptosis subtypes, high-fat diet feeding time and high-low ferroptosis score in GSE109345. **(B)** The relationship between ferroptosis subtypes, fibrosis stage and high-low ferroptosis score in GSE162694.





**Supplementary Figure 6**. Differential Analysis of Expression of Immune Checkpoint and Ferroptosis Marker Molecules Between Subtypes. **(A)** Differential expression of immune checkpoint genes between two clusters.**(B)** The correlation between different immune cells in MCPcounter. **(C)** Differential expression of ferroptosis marker molecules between two clusters. *p < 0.05, **p < 0.01, ***p < 0.001.





**Supplementary Figure 7**. Correlation Between ANXA2 Expression and Different Immune Cells. The expression of ANXA2 was positively correlated with NK cells **(A)**, endothelial cells **(B)** and fibroblasts **(C)**. The expression of ANXA2 was negatively correlated with monocytic lineage **(D)**, cytotoxic lymphocytes **(E)** and B lineage **(F)**.


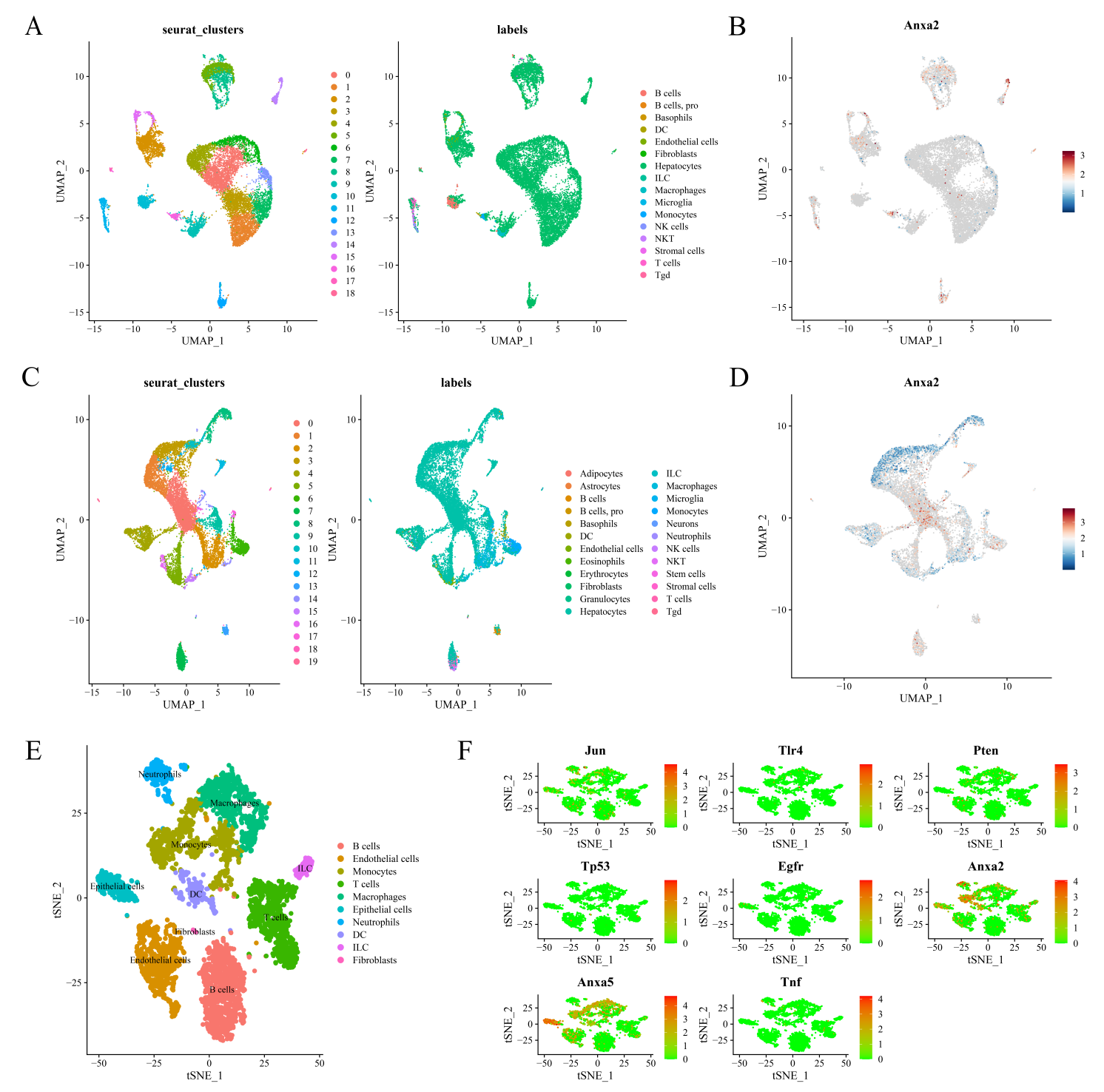


**Supplementary Figure 8.** Single-nucleus Analysis and Single-cell Analysis. **(A)** UMAP depicts cell clusters in a sample set of control samples (n=2), SingleR annotation of cell clusters. **(B)** The expression pattern of ANXA2 in control group. **(C)** UMAP depicts cell clusters in a sample set of NAFLD samples (n=2), SingleR annotation of cell clusters. **(D)** The expression pattern of ANXA2 in NAFLD group. **(E)** T-SNE visualization of cell types in liver biopsies from healthy (n = 4) and Diet-induced obesity (DIO)-NASH (n = 2) mice. Cells were annotated as B cells, Endothelial cells, Monocytes, T cells, Macrophages, Epithelial cells, Neutrophils, DCs, ILCs, Fibroblasts according to the expression of spectral markers. **(F)** Expression pattern of 8 score genes at the single-cell level. Red for expression, green for non-expression.





**Supplementary Figure 9. (A)** Western blot analysis. Compared with the control group, ANXA2 levels were significantly lower in the si-ANXA2 group. **(B)** Quantitative histogram showing the significant knockdown of ANXA2. **(C)** Histogram of ferrous ion levels in HepG2 cells in each group. **(D)** Histogram of lipid peroxidation levels in HepG2 cells in each group.


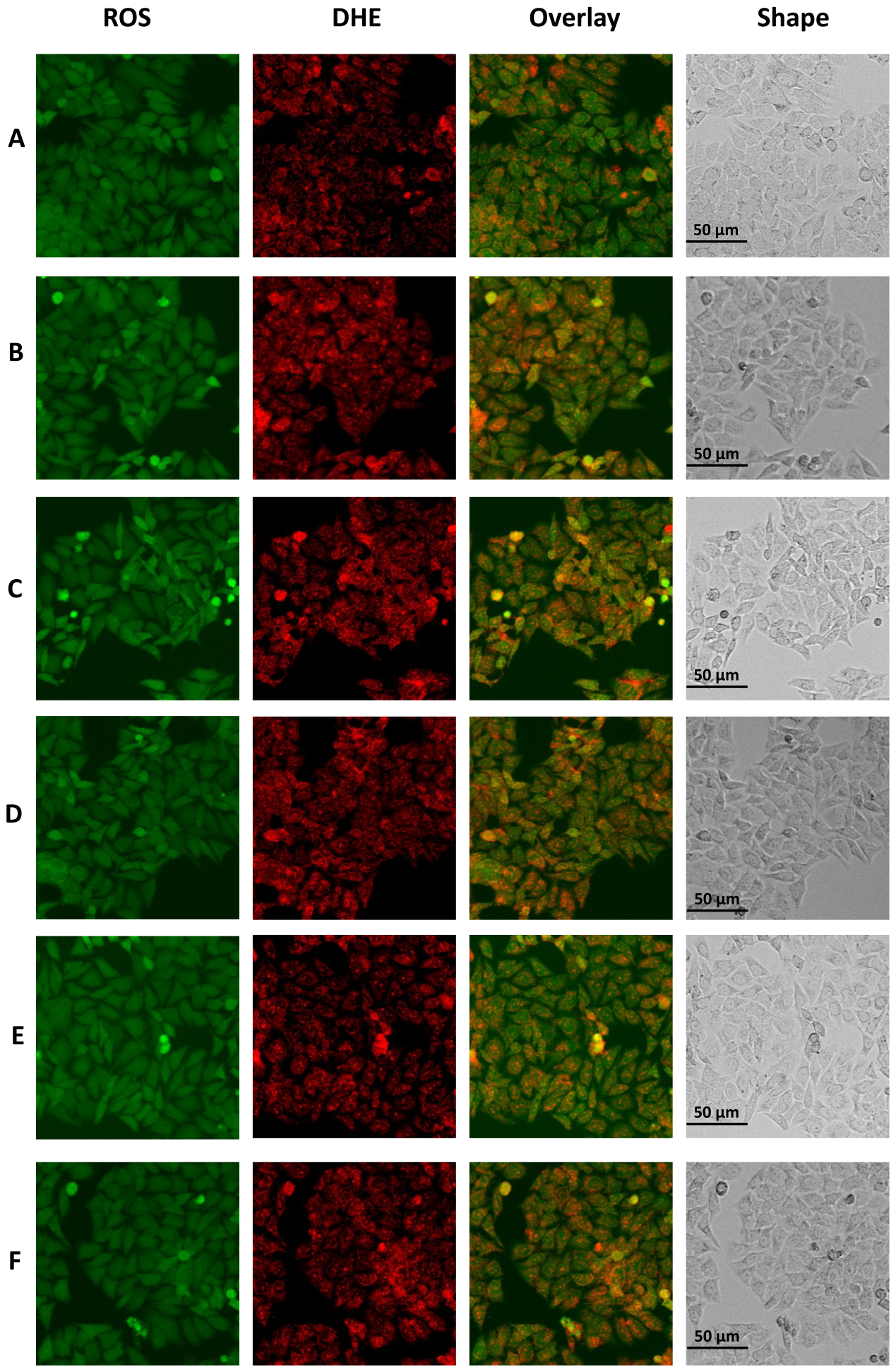


**Supplementary Figure 10**. The effect of ANXA2 on ROS and DHE levels in cells. **(A)** Normal control HepG2 cells; **(B)** HepG2 cells treated erastin (20 μM) for 24 h; **(C)** HepG2 cells co-cultured with siRNA control for 48 h and then treated erastin (20 μM) for 24 h; **(D)** HepG2 cells co-cultured with siANXA2-1 control for 48 h and then treated erastin (20 μM) for 24 h; **(E)** HepG2 cells co-cultured with siANXA2-2 control for 48 h and then treated erastin (20 μM) for 24 h; (**F)** HepG2 cells treated erastin (20 μM) and Ferrostatin-1 (2 μM) for 24 h. (The scale bar indicates 100 μm length, cells were incubated with DCFH-DA and DHE for 30 min to label intracellular ROS and superoxide anion, respectively)

**Supplementary Table S1**. Detailed information of the GEO datasets.

| GEO Accession | Platform | Number of normal sample | Number of NAFLD sample | Total |
| --- | --- | --- | --- | --- |
| GSE40481 | GPL6887 | 27 | 24 | 51 |
| GSE109345 | GPL19057 | 30 | 48 | 78 |
| GSE162694 | GPL21290 | 66 | 77 | 143 |
| GSE158241 | GPL19057 | 4 | 2 | 6 |
| GSE225381 | GPL24247 | 2 | 2 | 4 |
| In all | - | 129 | 153 | 282 |

**Supplementary Table S2**. Up-regulated and down-regulated differential genes in GSE40481.

| **ID** | **LogFC** | **AveExpr** | **t** | **P.Value** | **adj.P.Val** | **B** |
| --- | --- | --- | --- | --- | --- | --- |
| CFD | 4.864910866 | 10.59761541 | 19.4641201 | 2.01E-25 | 1.37E-21 | 47.12904238 |
| LCN2 | 2.633370222 | 10.09542449 | 11.5850701 | 5.70E-16 | 2.36E-13 | 26.13358638 |
| UBD | 2.193556269 | 8.810137255 | 13.54891313 | 1.26E-18 | 1.56E-15 | 32.10371194 |
| GSTA5 | 1.988672662 | 8.603782922 | 8.392099087 | 3.26E-11 | 3.19E-09 | 15.3688637 |
| HLA-DRB5 | 1.947858958 | 10.54223818 | 13.35066998 | 2.28E-18 | 2.60E-15 | 31.52475199 |
| HLA-DQB1 | 1.857351028 | 10.96057976 | 13.6516998 | 9.27E-19 | 1.27E-15 | 32.40182071 |
| CD74 | 1.703575343 | 10.63700592 | 12.60738552 | 2.22E-17 | 1.64E-14 | 29.30676935 |
| CIDEA | 1.669041287 | 8.162211824 | 4.591682647 | 2.86E-05 | 0.000564267 | 1.972122613 |
| SAA1 | 1.612448463 | 10.68827565 | 9.857731673 | 1.85E-13 | 3.24E-11 | 20.45736036 |
| LGALS3 | 1.568286375 | 10.39754245 | 12.74744536 | 1.44E-17 | 1.23E-14 | 29.73044045 |
| HLA-DQA1 | 1.481238662 | 9.315200451 | 6.000243067 | 1.97E-07 | 7.13E-06 | 6.812546287 |
| KRT23 | 1.392542222 | 10.54847376 | 12.29236452 | 5.95E-17 | 3.53E-14 | 28.34410379 |
| HLA-DMA | 1.372186495 | 8.998393118 | 11.17789291 | 2.15E-15 | 7.00E-13 | 24.83044272 |
| ANXA2 | 1.360936565 | 10.96599592 | 9.789419222 | 2.34E-13 | 3.86E-11 | 20.22522488 |
| CXCL2 | 1.331012755 | 9.810309647 | 6.754809441 | 1.26E-08 | 6.31E-07 | 9.509109227 |
| HLA-DMB | 1.275473787 | 8.736217588 | 12.55917975 | 2.58E-17 | 1.76E-14 | 29.16033333 |
| AATK | 1.22943087 | 9.805207667 | 6.704365061 | 1.52E-08 | 7.37E-07 | 9.327978161 |
| FCER1G | 1.20008312 | 10.32276729 | 9.652272732 | 3.77E-13 | 5.92E-11 | 19.75755401 |
| CYBA | 1.176819463 | 9.991620431 | 9.491020712 | 6.61E-13 | 1.00E-10 | 19.20496269 |
| ABCG5 | 1.146208972 | 13.1764589 | 14.59639932 | 5.90E-20 | 1.01E-16 | 35.07610729 |
| FPR2 | 1.139976009 | 8.944052373 | 8.383440837 | 3.36E-11 | 3.26E-09 | 15.338215 |
| CD274 | 1.102079991 | 8.985496804 | 9.703972214 | 3.15E-13 | 5.06E-11 | 19.9341027 |
| GDF15 | 1.08597213 | 9.225820745 | 4.976728139 | 7.58E-06 | 0.000177632 | 3.255319032 |
| C1QB | 1.039960403 | 11.07658269 | 9.257672879 | 1.50E-12 | 2.07E-10 | 18.40028061 |
| TLR2 | 1.033786319 | 8.362834235 | 13.24025018 | 3.19E-18 | 3.35E-15 | 31.19998338 |
| CCL5 | 1.031728653 | 8.219922255 | 6.250580033 | 7.95E-08 | 3.14E-06 | 7.703195419 |
| FCGR3A | 1.027219611 | 10.48425602 | 9.973873248 | 1.24E-13 | 2.23E-11 | 20.85076748 |
| BCL2A1 | 1.019146704 | 8.354244078 | 9.975778564 | 1.23E-13 | 2.23E-11 | 20.85720804 |
| LGALS1 | 1.002876431 | 9.787043961 | 3.704472213 | 0.000516984 | 0.006566075 | -0.801432656 |
| MMD2 | -1.041597343 | 9.133961471 | -5.550684206 | 9.97E-07 | 2.97E-05 | 5.229713608 |
| TM7SF2 | -1.067654653 | 8.742713059 | -18.04489401 | 6.10E-24 | 2.78E-20 | 43.89231314 |
| TFF3 | -1.117485597 | 8.02450898 | -6.218252891 | 8.94E-08 | 3.46E-06 | 7.587897178 |
| FDPS | -1.121701102 | 8.98124951 | -11.57112957 | 5.96E-16 | 2.39E-13 | 26.08933787 |
| STARD4 | -1.123543968 | 9.90886102 | -10.49073624 | 2.13E-14 | 5.63E-12 | 22.58170088 |
| MSMO1 | -1.129211861 | 9.489442294 | -10.46961501 | 2.29E-14 | 5.90E-12 | 22.51161785 |
| UGT1A4 | -1.194234778 | 8.530489941 | -13.16478731 | 4.01E-18 | 3.91E-15 | 30.97708458 |
| CYP51A1 | -1.252923042 | 8.809802824 | -12.46915132 | 3.42E-17 | 2.22E-14 | 28.88600497 |
| CYP17A1 | -2.768322125 | 9.588226176 | -21.56457915 | 1.79E-27 | 2.45E-23 | 51.5523261 |

**Supplementary Table S3**. Characterized genes under LASSO, RF,SVM, Boruta analysis.

| **LASSO** | **RF** | **SVM** | **Boruta(top10 of 45)** |
| --- | --- | --- | --- |
| SREBF1 | SQLE | SQLE | SQLE |
| ANXA2 | SLC2A6 | SLC2A6 | SLC2A6 |
| MYH10 | ANXA2 | ANXA2 | CKB |
| TP53 | CKB | CD44 | ANXA2 |
| TNFAIP3 | - | ACSL5 | LGALS3 |
| FADS2 | - | CKB | CD44 |
| WWTR1 | - | LGALS3 | TNF |
| LCN2 | - | PRKCB | TNF |
| SQLE | - | PCSK9 | PRKCB |
| PLTP | - | CAPG | PCSK9 |
| IRF1 | - | - | - |
| LGALS3 | - | - | - |

**Supplementary Table S4**. The set of genes progressively up-regulated in the time series analysis.The results of GSE40481 and the intersection of GSE40481, GSE109345 and GSE162694 are shown separately.

| **GSE40481** | - | **Intersection** | - |
| --- | --- | --- | --- |
| ATG7 | MYH9 | ATF3 | AR |
| GABARAPL1 | MYH10 | SLC1A4 | TLR4 |
| ATF3 | TRIM28 | HSPB1 | SRXN1 |
| ATM | PFN2 | JUN | - |
| SLC1A4 | RUFY1 | EMP1 | - |
| GDF15 | AR | CYGB | - |
| TUBE1 | SMARCA4 | CX3CL1 | - |
| CXCL2 | MYO9B | LASP1 | - |
| HSPB1 | MAGED2 | PDGFRB | - |
| ISCU | DUOX1 | ANXA2 | - |
| JUN | TLR4 | ANXA5 | - |
| PRNP | SRXN1 | GNB1 | - |
| SLC11A2 | SQSTM1 | MYH9 | - |
| EMP1 | PLTP | SMARCA4 | - |
| SREBF1 | CDH2 | MYO9B | - |
| USP35 | EEF1A1 | MAGED2 | - |
| CYGB | CFTR | SQSTM1 | - |
| CX3CL1 | ANXA2 | GDF15 | - |
| LASP1 | ANXA5 | SLC11A2 | - |
| PDGFRB | GNB1 | MYH10 | - |
